# Cyclodextrin-based Schiff base pro-fragrances: Synthesis and release studies

**DOI:** 10.3762/bjoc.18.140

**Published:** 2022-09-28

**Authors:** Attila Palágyi, Jindřich Jindřich, Juraj Dian, Sophie Fourmentin

**Affiliations:** 1 Department of Organic Chemistry, Faculty of Science, Charles University, Hlavova 8, CZ-128 43 Prague, Czech Republic; 2 Department of Chemical Physics and Optics, Faculty of Mathematics and Physics, Charles University, Ke Karlovu 3, 121 16, Prague 2, Czech Republic; 3 Department of Analytical Chemistry, Faculty of Science, Charles University, Hlavova 8, CZ-128 43, Prague, Czech Republic; 4 Unité de Chimie Environnementale et Interactions sur le Vivant (UCEIV), UR 4492 SFR Condorcet FR CNRS 3417, Université du Littoral-Côte d'Opale (ULCO), Dunkerque, France

**Keywords:** aldehyde, controlled release, cyclodextrin, imine, kinetics, pro-fragrance, Schiff base

## Abstract

A simple method for the preparation of β-cyclodextrin derivatives containing covalently bonded aldehydes via an imine bond was developed and used to prepare a series of derivatives from 6^I^-amino-6^I^-deoxy-β-cyclodextrin and the following volatile aldehydes – cinnamaldehyde, cyclamen aldehyde, lilial, benzaldehyde, anisaldehyde, vanillin, hexanal, heptanal, citral, and 5-methylfurfural. Subsequently, the rate of release of the volatile compound from selected pro-fragrances, as a function of the environment (solvent, pH), was studied by ^1^H NMR spectroscopy (for benzaldehyde) and static headspace-gas chromatography (for benzaldehyde, heptanal, and 5-methylfurfural). The aldehyde release rate from the imine was shown to depend substantially on the pH from the solution and the air humidity from the solid state.

## Introduction

The fragrance and flavor industry is one of the most intensively developing sectors of the chemical industry. Encapsulation techniques are widely used in both food and cosmetic industries to control the delivery of the encapsulated guest molecules and protect those agents from environmental degradation [1–2]. Cyclodextrins (CDs) serve as one of the simplest encapsulating systems. CDs are cyclic oligosaccharides composed of 1→4 linked α-ᴅ-glucopyranose units (6, 7, and 8 for the most common α-, β-, and γ-CD). CDs are well known for their hydrophilic outer surface and hydrophobic cavity. This cavity can encapsulate another lipophilic guest molecule and thus form an inclusion complex [3–4]. This phenomenon is reversible and leads to an equilibrium between encapsulated and free guest.

A staggering number of inclusion complexes of CDs with various organic molecules have been described so far. It was proved that the complexation of volatile organic compounds, such as aldehydes, into the CD’s cavity reduces the volatility and increases the solubility and bioavailability of these compounds [5–14]. The release of the included molecule from the CD’s cavity takes from minutes to hours, depending on the environmental conditions as well as on the structure of the molecule. The prolongation of the release time of the complexed compounds would make significant progress in fragrance delivery as well as in an odor and flavor control; it could be used to improve the stabilization, quality, efficiency and persistence of repellents, disinfectants, perfumes, laundry detergents and flavoring agents [15–16].

**Figure 1:**
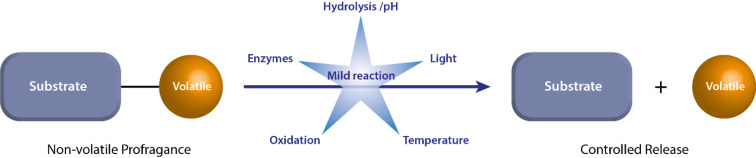
Concept of the controlled release of volatile organic molecules from pro-fragrances [18].

Another strategy to prolong the longevity of the fragrance and, as an additional benefit, to increase the stability of labile compounds is to prepare pro-fragrances in analogy with the concept of pro-drugs developed for pharmaceutical applications. The fragrance is linked covalently to a substrate that will release the fragrance under defined chemical conditions. For applications in the fragrance and flavor industry, the covalent bond that links the fragrance to its substrate must be cleaved under environmental conditions found in everyday life, and the substrate should be non-volatile and non-toxic [17]. Typical triggers that may be used for mild chemical reactions are temperature, enzymatic or pH-dependent hydrolysis, oxidation, or light (Figure 1) [18]. Different substrates were used to synthesize pro-fragrances, like polymers [19], ionic liquids [20], rotaxanes [21], or saccharides [22].

To the best of our knowledge, no studies investigate the use of CDs as substrate. Linking the fragrance to CDs could present an advantage compared to simple encapsulation and to other substrates as they will not only ensure very low volatility of the obtained pro-fragrance but also be able to encapsulate the guest after the cleavage of the covalent bond, leading to a two-in-one system. Indeed, encapsulation in CDs is known to modify and/or improve the physical and chemical properties of the included guest, ensuring the protection of labile molecules from photodegradation or oxidation [3,6,23]. Moreover, many CD derivatives are available, opening the possibility of using different chemical functions (ester, imine…) to attach the fragrance covalently with various functional groups. Additionally, CDs already occur in many daily products [16] and fulfill the requirements of biocompatibility and cost for designing pro-fragrances that could find applications in the flavor and fragrance industry [18].

In this article, we describe the synthesis of ten pro-fragrances – Schiff bases prepared from amino-β-cyclodextrin and common volatile aldehydes. Aldehydes constitute a prominent class of molecules broadly used as food product additives and are also key components of perfumes [24]. They were therefore chosen as an example of flavor compounds. The imine bond was chosen for its relative stability; on the other hand, it can be readily hydrolyzed forming the starting non-volatile amine and releasing the aldehyde. The kinetics of the aldehyde release was studied by ^1^H NMR techniques in buffers with different pH values. The aldehyde release itself from the buffers and by humidity was followed by static headspace-gas chromatography (SH-GC).

## Results and Discussion

### Synthesis of pro-fragrances

6^I^-Amino-6^I^-deoxy-β-cyclodextrin (amino-β-CD, **1**) was chosen as the most appropriate amino-cyclodextrin derivative due to its easiest accessibility; besides, β-CD forms usually the strongest inclusion complexes compared to α-CD and γ-CD. The amino-β-CD was prepared according to published procedures [25–27].

**Figure 2:**
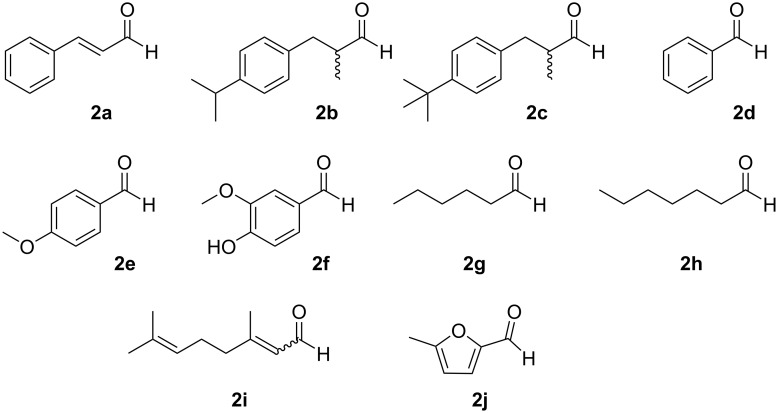
The common commercially available aldehydes used for binding to amino-β-CD (**1**): *trans*-cinnamaldehyde (**2a**), cyclamen aldehyde (**2b**), lilial (**2c**), benzaldehyde (**2d**), anisaldehyde (**2e**), vanillin (**2f**), hexanal (**2g**), heptanal (**2h**), citral (**2i**), 5-methylfurfural (**2j**).

Common commercially available volatile aldehydes **2a–j** (Figure 2) were chosen as the aldehyde reactants. Some of the aldehydes were mixtures of isomers, but it was acceptable for our intended purpose – to study the controlled release of the aldehydes, i.e., volatile organic compounds (VOCs).

The aldehyde selected for the optimization of reaction conditions was cinnamaldehyde (**2a**), as it is strongly UV absorbing, i.e., easy to follow by TLC. But in the end, the reaction progress was monitored by MS due to the fast hydrolysis of the imine even during the chromatography on TLC plates. Methanol was chosen as a solvent because it dissolves the aldehydes well and sufficiently (≈5 mg/mL) amine **1**.

At first, we studied the influence of adding hygroscopic salts (MgSO_4_, LiClO_4_, or Ca(ClO_4_)_2_), triethyl orthoformate, or activated molecular sieves to the reaction. These compounds could function as desiccants removing the water formed during the reaction, as well as catalysts; nevertheless, the conversion to the final imine needed, in all cases, a large excess of an aldehyde.

**Scheme 1:**
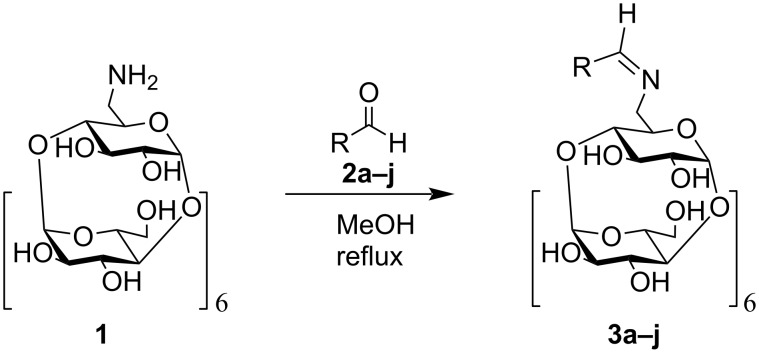
Preparation of the Schiff bases from amino-β-CD **1** and aldehydes **2a**–**j**. Yields: **3a**, 96%; **3b**, 83%; **3c**, 91%; **3d**, 86%; **3e**, 82%; **3f**, 96%; **3g**, 89%; **3h**, 97%; **3i**, 80%; **3j**, 83%.

Finally, the best reaction conditions for the large-scale preparation of target compounds proved to be just refluxing of amine **1** with up to 30-fold excess of the aldehydes **2a**–**j** in methanol (Scheme 1), which afforded the final imines **3a**–**j** in high yields (80–97%).

This method allows for easy recovery of the unreacted aldehyde as well as separation of the product (just by extraction by hexane and drying under reduced pressure) without its decomposition. The structure of the final imines was confirmed by ^1^H NMR, ^13^C NMR, ESI–MS, and the release of the VOCs was next studied by ^1^H NMR and SH-GC for selected imino-β-CD.

The prepared imines could also be mixtures of *E*/*Z* isomers, but no attempts were made to isolate them for the reason metioned above. Also, the prepared imines proved to be difficult to purify by chromatographic methods due to their low stability towards hydrolysis.

### Kinetic studies of the imine hydrolysis by NMR

For the kinetic studies of the hydrolysis of imino-CDs, we selected the pro-fragrance **3d** made from the most common aldehyde – benzaldehyde. The release of the benzaldehyde (**2d**) was studied by ^1^H NMR spectroscopy. Aqueous 0.1 M phosphate buffer solutions of pH 1.08, 2.00, 3.00, 4.00, 5.00, 6.00, 7.00, 8.00, 9.00, 10.00 11.00, 12.00, and 12.80 were prepared (see Experimental section) using deuterium oxide instead of water to facilitate NMR spectroscopy experiments. Because of the low solubility of the pro-fragrance in water, it had to be dissolved in deuterated dimethyl sulfoxide. Then, the buffer solution was added and mixed in a 1:1 (v/v) ratio just before starting the measurements. The samples were kept at ambient temperature (20–25 °C).

All measurements for a given pH value were repeated at least three times with about 10 mg of the pro-fragrance **3d**. For the pH in the range 1.08–4.00, the samples were taken and measured in intervals from 2 min up to 24 h for several days, the remaining samples with higher pH were measured in 2 h to several day intervals for up to three months. The integrals of the signal at 8.30 ppm (hydrogen of the imine group of Schiff base) were compared to the integral of the signal belonging to the same proton of the starting compound **3d** measured in DMSO-*d_6_* (without a buffer) used as a blank.

We note that the experiment was conducted in a closed system with a 50% content of DMSO-*d*_6_ to enable dissolution of the pro-fragrance, which influenced the equilibrium between dissociation and formation reactions. The integrals of the imine group signal at 8.30 ppm measured at various time intervals were depicted as a function of time for all the samples with pH from 3 to 12.8. Data were fitted by a nonlinear regression method (Levenberg–Marquardt algorithm) using both mono- and double-exponential functions (Microcal Origin software). In most cases, the double-exponentials described the observed behavior much better (see Supporting Information File 1, Figures S1–S11). Experimental data were fitted with the double-exponential function of the form:


[1] $$I=I_{0}+I_{1}e^{-((t-t_{0})/t_{1})}+I_{2}e^{-((t-t_{0})/t_{2})}$$

where *I*_0_, *I*_1,_ and *I*_2_ are fitting parameters corresponding to the integrated areas of the background and corresponding time components, *t*_0_ is a parameter related to the correction of zero time, and *t*_1_ and *t*_2_ are time constants of the slow and fast components of the double-exponential decay curve.

**Figure 3:**
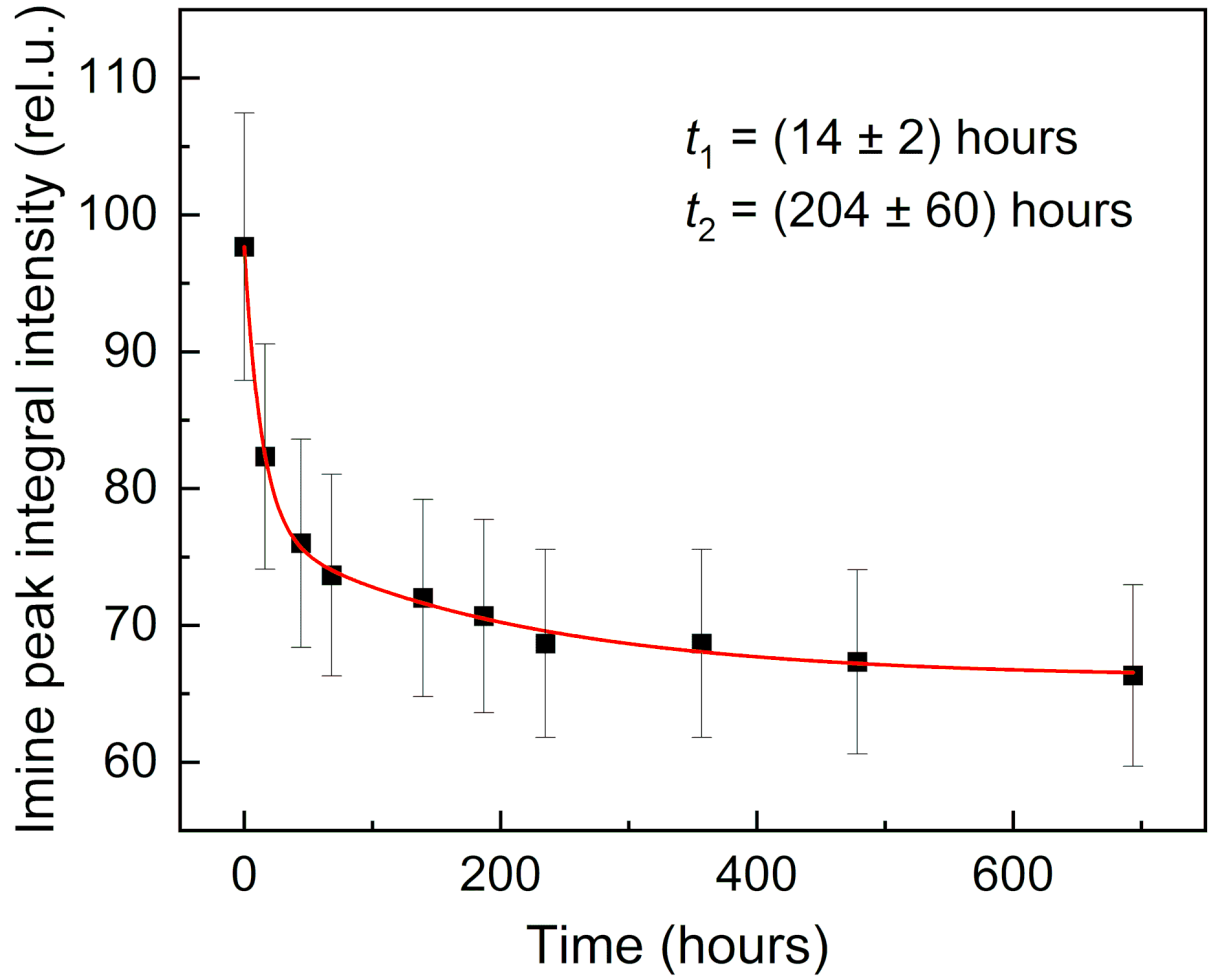
The integrals of the imine group proton signal at 8.30 ppm in the acquired ^1^H NMR spectrum of compound **3d** at pH 9 as a function of time. Time constants *t*_1_ and *t*_2_ correspond to the fast and slow components of the double-exponential decay function. The red line is the corresponding fit of the experimental data by Equation 1.

A typical example of the time decay of the integral of the imine signal area for the sample at pH 9 is depicted in Figure 3.

**Figure 4:**
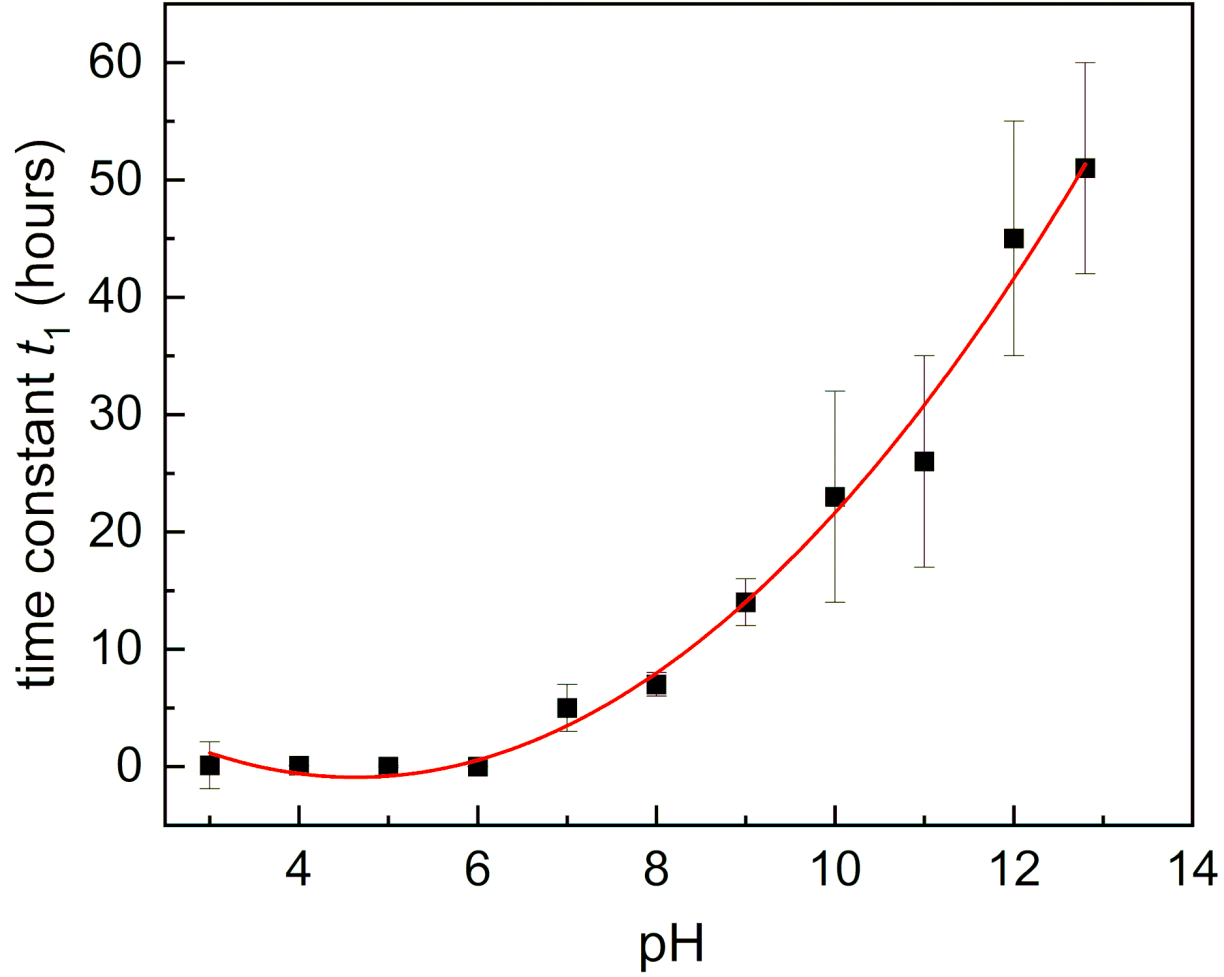
pH behavior of the time constant *t*_1_ of the fast component in the time evolution of the NMR signal of the imine group of the compound **3d**. Error bars correspond to the regression error; red line corresponds to the polynomial fit of the experimental data.

For lower pH values (pH 1 and 2), the hydrolysis was too fast to be investigated by ^1^H NMR spectroscopy; the experimental data are summarized in Table S1 in Supporting Information File 1. To visualize the pH dependence of the time decay of the integral of the imine group, we chose the time constant of the fast component *t*_1_ and depicted it as a function of pH (Figure 4).

Figure 4 shows that the pH behavior of *t*_1_ component is nearly monotonic (except for low pH values). A substantial difference is seen between acidic conditions, under which the imino-β-CD is unstable, and the benzaldehyde releases fast over the time course investigated, and basic conditions, under which the benzaldehyde is released slowly. In all cases, equilibrium between the hydrolyzed and the non-hydrolyzed pro-fragrance is obtained – the more acidic the solution is, the equilibrium is reached faster, and the imine is hydrolyzed to a higher degree. For pH 1 and 2 the hydrolysis degree is about 90%, which decreases with the pH rising.

It has to be noted that during the NMR studies, no degradation of the cyclodextrin was detected with the available methods. At pH of 11.00, 12.00 and 12.80, a decrease and later (in the case of pH 12.80) a total disappearing of the hydrogen signal of the aldehyde group of benzaldehyde at 10.06 ppm was observed due to Cannizaro’s reaction. At pH 12.80, the benzaldehyde was fully converted to benzyl alcohol and benzoic acid after 5 days.

### Static headspace analysis

We first determined the Henry law constants and the formation constants with β-CD of three selected aldehydes (Table 1). As can be seen from Table 1, the studied aldehydes exhibit Henry law constant <1. The obtained formation constants are in good agreement with values obtained for aromatic or linear flavors [28].

**Table 1:** Henry law constants and formation constants with β-CD (M^−1^) of the three selected aldehydes (30 °C, aqueous solution).

aldehyde	benzaldehyde (**2d**)	heptanal (**2h**)	5-methylfurfural (**2j**)

*H*_c_	0.10	0.16	0.02
*K*_f_	103	452	61

**Figure 5:**
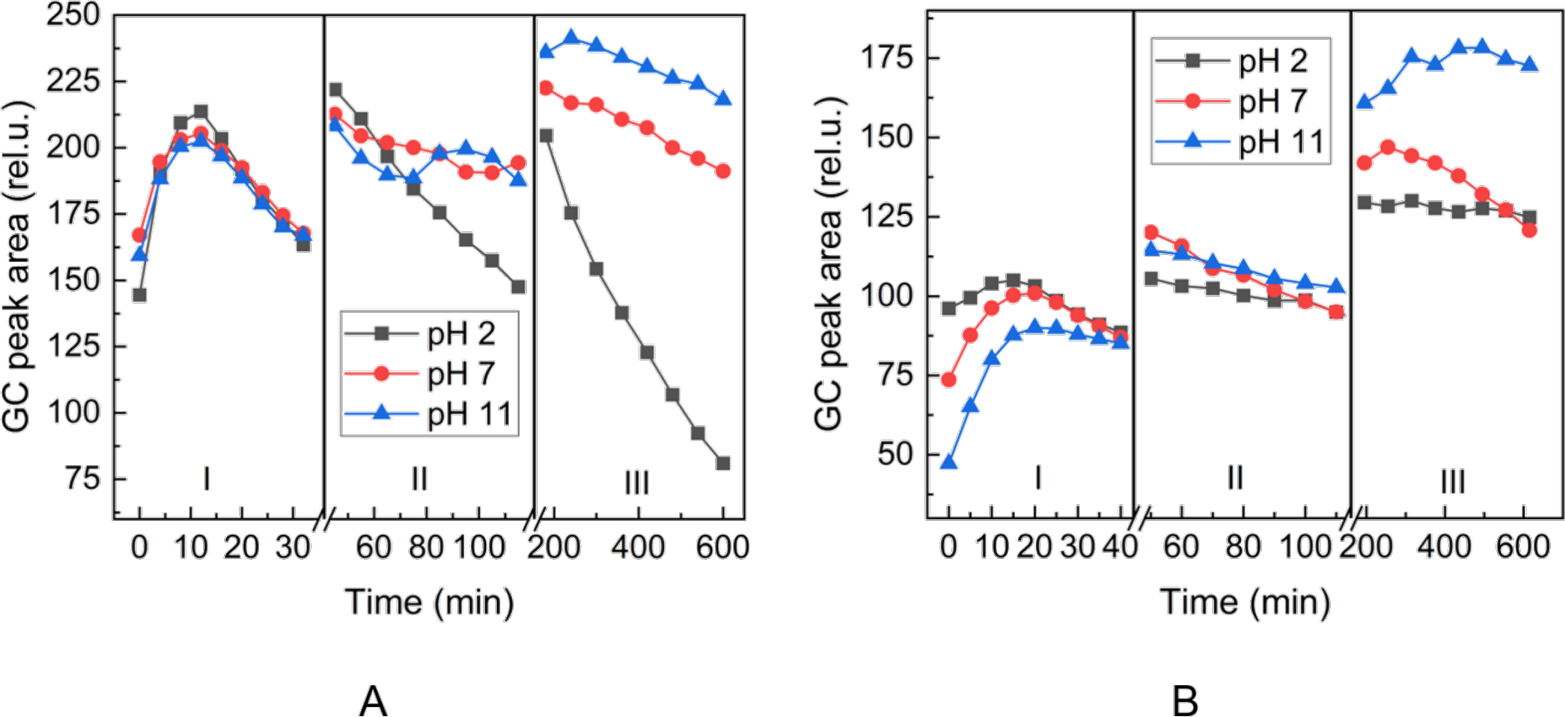
Time evolution of the headspace concentration of heptanal (**2h**) after successive extractions from the gaseous phase above (A): heptanal (**2h**) in aqueous phosphate buffers and (B): heptanylimino-β-CD (**3h**) in aqueous phosphate buffers. Sampling intervals in the corresponding regions: I – 4 min (A) or 5 min (B), II – 10 min, III – 60 min.

**Figure 6:**
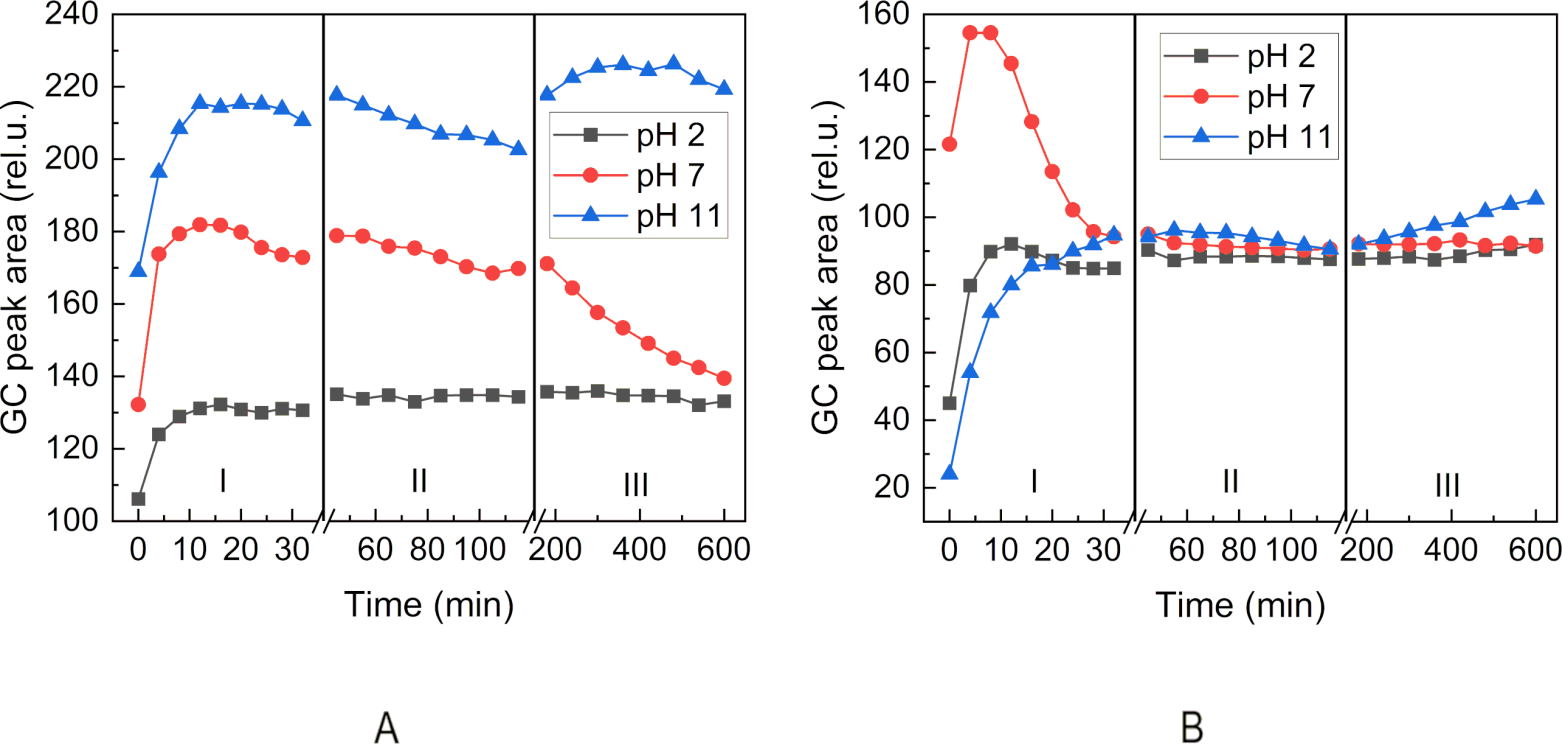
Time evolution of the headspace concentration of benzaldehyde (**2d**) after successive extractions from the gaseous phase above (A): benzaldehyde (**2d**) in aqueous phosphate buffers and (B): benzylimino-β-CD (**3d**) in aqueous phosphate buffers. Sampling intervals in the corresponding regions: I – 4 min, II – 10 min, III – 60 min.

**Figure 7:**
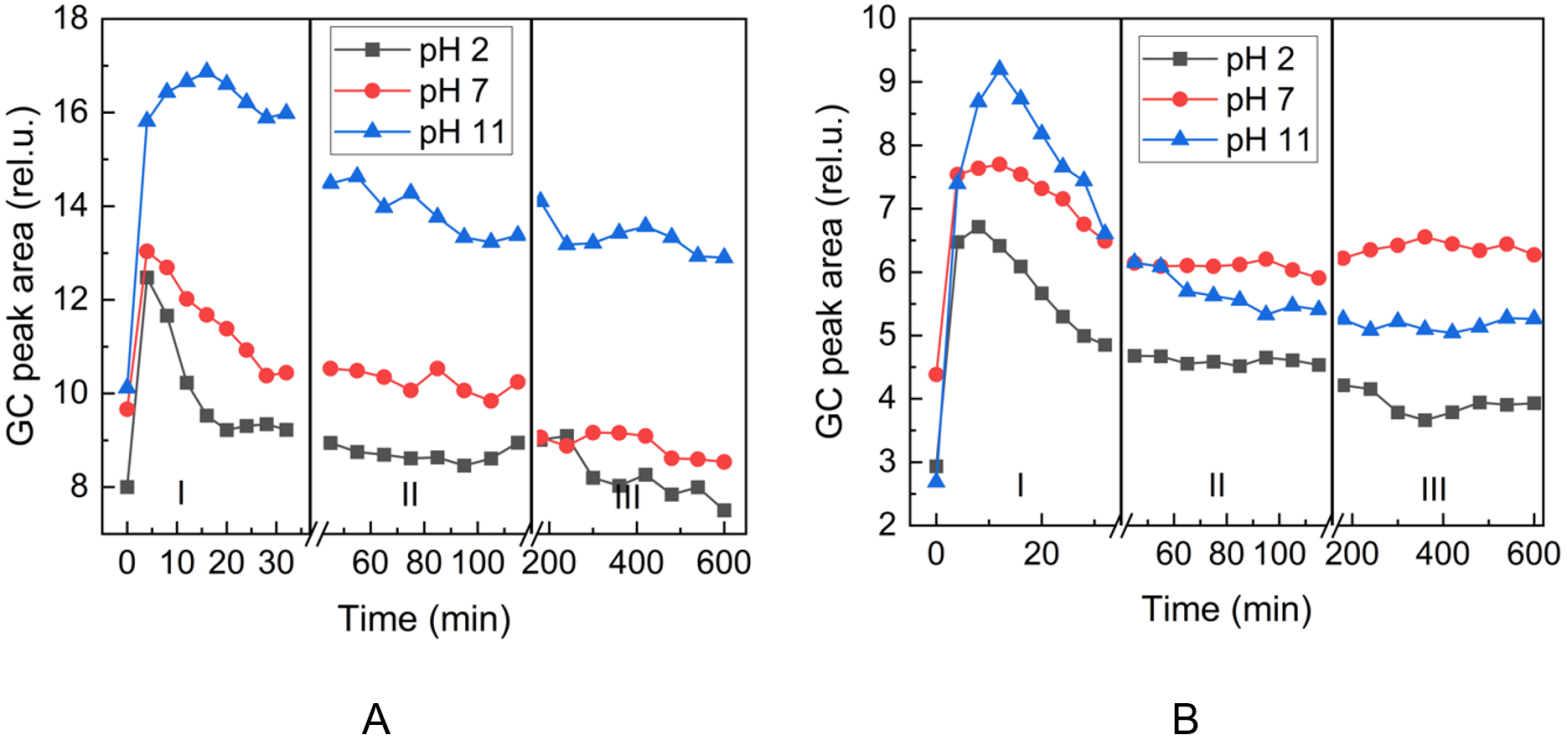
Time evolution of the headspace concentration of 5-methylfurfural (**2j**) after successive extractions from the gaseous phase above (A): 5-methylfurfural (**2j**) in aqueous phosphate buffers and (B): 5-methylfurfurylimino-β-CD (**3j**) in aqueous phosphate buffers. Sampling intervals in the corresponding regions: I – 4 min, II – 10 min, III – 60 min.

Multiple headspace extraction experiments were performed to follow the release of the aldehydes. This technique was already successfully used to follow the release of flavor in solution or solid state [29–31]. The aldehyde release was studied using three different sampling time intervals corresponding to three time regions I, II, and III, in Figures 5–7. This experiment mode enabled better observation of the trends in the change of the aldehyde headspace concentrations at different sampling times and pH.

First, we follow the release of heptanal in aqueous buffer solutions (Figure 5A). We can observe a decrease of the area due to the depletion of the aldehyde in the aqueous solution after successive extractions, while the release of heptanal from heptanylimino-β-CD is more sustained (Figure 5B). Similar behavior at various pH conditions could be observed in the case of the release of benzaldehyde (**2d**) from the phosphate buffer (Figure 6A) and benzylimino-β-CD (**3d**, Figure 6B) and 5-methylfurfural (**2j**) from the phosphate buffer (Figure 7A) and 5-methylfurfurylimino-β-CD (**3j**, Figure 7B).

Figures 5–7 show that the aldehyde release from the β-CD imino derivatives was slower than the release from the corresponding aldehyde aqueous phosphate buffers. This observation is consistent with the role of the physicochemical barrier that slows down the release of aldehyde due to the supramolecular interaction between aldehyde and β-CD cavity.

It follows from Figures 5–7 that heptanal (**2h**) is released faster with lower pH values, whereas benzaldehyde (**2d**) and 5-methylfurfural (**2j**) show opposite trends. It can be attributed to the lower boiling point (153 °C) and lower hydrophilicity (log*P* 2.5) of the aliphatic heptanal compared to the two aromatic aldehydes with higher boiling points (179 °C, 187 °C) and more than an order of magnitude higher hydrophilicity (log*P* 1.48, 0.654) (physical properties values taken from chemspider.com).

**Figure 8:**
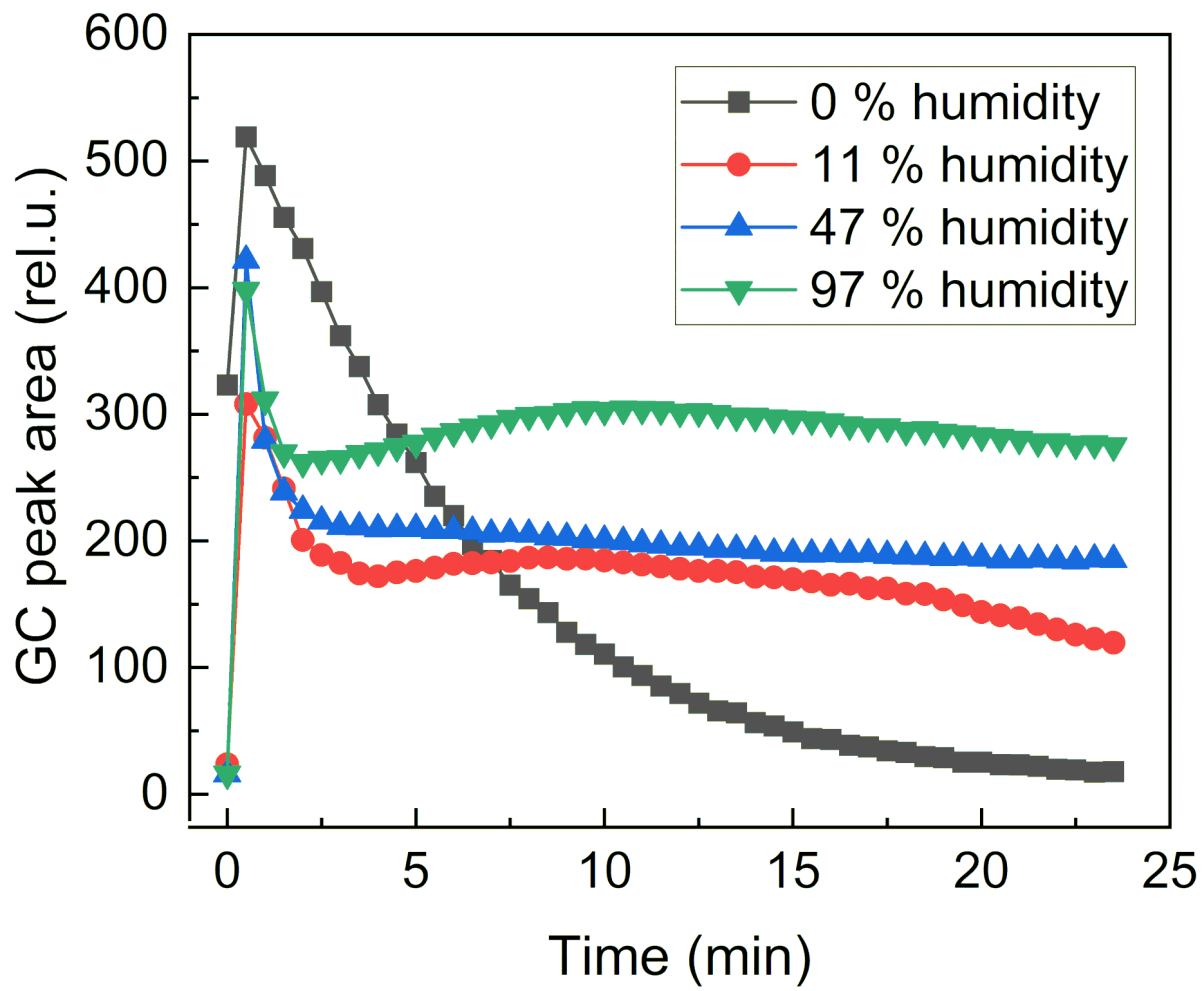
Evolution of the area of 5-methylfurfural released from 5-methylfurfurylimino-β-CD (**3j**) upon time after successive extractions of the gaseous phase at different RH% values.

Finally, the release of 5-methylfurfural (**2j**) from 5-methylfurfurylimino-β-CD (**3j**) was monitored at different humidity (RH%). In this case, a powder of 5-methylfurfurylimino-β-CD (**3j**) was placed in a small vial and exposed to different RH%. As we can see from Figure 8, humidity is necessary to trigger the release of the volatile aldehyde as the amount of released volatile increased with the RH%. In this experiment, we also observed a sustained release of the volatile compound from the imino-β-CD.

## Conclusion

Here a new family of pro-fragrances using CD as substrate was prepared for the first time. A general, simple, and high-yielding method for their preparation was developed – Shiff base pro-fragrance is formed by refluxing amino-β-CD with an excess of a volatile aldehyde in methanol and purified just by extraction. The use of CD is interesting as it acts as a double barrier carrier for fragrance aldehyde molecules. We illustrated that CD-based pro-fragrances combine a chemical barrier, as the aldehydes are linked to the CD via imine bond, and a physicochemical (supramolecular) barrier, as we established that aldehydes formed inclusion complexes in aqueous solution with β-CD. We confirmed from the NMR kinetic study of the imine bond decomposition that Shiff base hydrolysis is very fast in acidic conditions and slows down when going to higher pH values. Multiple headspace extraction experiments revealed the role of pH and the presence of supramolecular interaction between aldehyde and β-CD on the rate of aldehyde release from the system. Sustained release of the aldehyde was demonstrated both in aqueous solutions and from a solid state upon humidity exposure.

## Experimental

### Instruments, general methods, and chemicals

^1^H NMR, ^13^C NMR, 2D NMR (H,H-COSY, HSQC, and HMBC) were measured on Bruker AVANCE III 600 MHz (600.17 MHz for ^1^H, 150.04 MHz for ^13^C) and Varian ^UNITY^INOVA 400 (399,95 MHz for ^1^H and 100,58 MHz for ^13^C) spectrometers. For the kinetic studies, the ^1^H NMR spectra were acquired on a Varian VNMRS 300 spectrometer (300 MHz for ^1^H). DMSO-*d*_6_ and D_2_O were used as the solvents. The chemical shift values (δ) are given in ppm, and the values of the interaction constants (*J*) in Hz. Standard numbering for cyclodextrin’s glucose units and numbering with apostrophes for substituents were used to assign NMR signals.

Static headspace-gas chromatography measurements were conducted with an Agilent headspace autosampler. Henry law constants (*H*_c_) were determined as described previously [32–33]. Briefly, the same amount of aldehyde was added to vials containing different amounts of water. Using the phase ratio variation method as described by Kolb and Ettre [34], the values of *H*_c_ were determined by the relationship between the reciprocal chromatographic peak areas and the vapor–liquid volumetric ratio. The formation constant values were determined as described in previous works [28,35–36].

To study the release of aldehydes from aqueous buffered solutions, 10 mL of 0.1 M aqueous phosphate buffers were introduced into a 22 mL headspace vial, then 4 mg of compounds **3** were introduced in the vial, immediately sealed using silicone septa and aluminum foil, and analyzed by multiple headspace extraction at 30 °C. To evaluate the effect of humidity, 4 mg of compound **3** was put in a small (2 mL) vial, which was embedded into a 22 mL headspace vial containing 1 mL of saturated salt solution to obtain the given percentage of humidity at 60 °C and sealed using silicone septa and aluminum foil. Saturated K_2_SO_4_, KNO_3_, and LiCl salt solutions were used to obtain 97, 47, and 11% of humidity, respectively. The samples were then immediately analyzed by MHE at 60 °C. 1 mL of vapor from the above solution was withdrawn from the vial using a gas-tight syringe and injected directly into the chromatographic column via a transfer line (250 °C). Each sample was then analyzed by gas chromatography (Perkin Elmer Autosystem XL equipped with a flame-ionization detector using a DB624 column). The GC settings were set as follows: detector temperature, 250 °C; column temperature: 160 °C for benzaldehyde and 5-methylfurfural and 120 °C for heptanal. The retention times under the given conditions were 2.1 min for benzaldehyde (**2d**) and 5-methylfurfural (**2j**) and 3.1 min for heptanal (**2h**).

The mass spectra were measured by the Bruker ESQUIRE 3000 ES-ion trap and the samples were ionized using an electrospray technique (ESI). The samples were dissolved in methanol.

Specific optical rotation was measured by the Rudolph Research AUTOPOL™ III Polarimeter at 25 °C and at the wavelength of the sodium doublet. Specific optical rotation values ([α]_D_^25^) are given in 10^−1^ cm^2^·g^−1^.

For evaporation of the solvents, a rotary vacuum evaporator from Büchi was used at temperatures up to 50 °C and a Glass oven B-528 Kugelrohr from Büchi at temperatures up to 110 °C.

For thin-layer chromatography (TLC) DC-Alufolien Kieselgel 60 F265 (Merck, Darmstadt, Germany) silica gel plates were used. Carbonization in 50% sulfuric acid was used to detect the substances. An eluent mixture propanol/water/25% aqueous ammonia/ethyl acetate 6:3:1:1 (EM1) was used for TLC.

Anhydrous DMF was prepared by distillation with P_2_O_5_ at reduced pressure and was stored over molecular sieves 3 Å under argon atmosphere. Organic solvents were distilled before use. β-CD was purchased from WAKO Chemicals (Germany). Other reagents were purchased from common commercial sources and used without further purification (Sigma-Aldrich, Penta).

6^I^-*O*-*p*-Toluenesulfonyl-β-cyclodextrin, 6^I^-azido-6^I^-deoxy-β-cyclodextrin, and 6^I^-amino-6^I^-deoxy-β-cyclodextrin (**1**) were prepared according to the published procedures [25–27]. NMR spectra were in agreement with the literature.

### Kinetic studies of the imine hydrolysis by NMR

For the kinetic studies of the hydrolysis of imino-CDs, 6^I^-benzylideneamino-6^I^-deoxy-β-cyclodextrin (**3d**) was chosen. The release of the benzaldehyde was studied by ^1^H NMR spectroscopy. Aqueous 0.1 M phosphate buffer solutions of pH 1.08, 2.00, 3.00, 4.00, 5.00, 6.00, 7.00, 8.00, 9.00, 10.00 11.00, 12.00, and 12.80 were prepared by mixing 0.1 M solutions of H_3_PO_4_, KH_2_PO_4_, K_2_HPO_4,_ and K_3_PO_4_ in ratios given in Table 2 using deuterium oxide instead of distilled water to facilitate NMR spectroscopy experiments. The exact pH value was tuned with titration with the help of a pH meter. Because of the poor solubility of the pro-fragrance in water, it had to be dissolved in 0.5 mL of deuterated dimethyl sulfoxide. Then, the buffer solutions (0.5 mL) were added and mixed just before starting the measurements. The samples were kept at ambient temperature (20–25 °C). Every measurement for every pH value was repeated three times with about 10 mg of pro-fragrance **3d**. The measurement intervals of the benzaldehyde release by ^1^H NMR spectroscopy were 2 min to 24 h for several days for the buffers with pH values from 1.08 to 4.00. For the rest of the buffers, the intervals were 2 h to several days for up to 3 months (for basic pH). The integrals of the signal at 8.30 ppm, corresponding to the hydrogen of the imine group of the non-hydrolyzed Schiff base, were compared to the integral of the signal corresponding to the same proton of the pro-fragrance measured in DMSO-*d*_6_ (without buffer) used as a blank.

**Table 2:** Ratios of 0.1 M aqueous phosphates solutions used to prepare buffers of the specified pH.

pH	H_3_PO_4_	KH_2_PO_4_	K_2_HPO_4_	K_3_PO_4_

1.08	1	–	–	–
2.00	1	–	1	–
3.00	dropwise	1	–	–
4.00	–	1	dropwise	–
5.00	–	1	dropwise	–
6.00	–	10	1	–
7.00	–	15	5	3
8.00	–	3	17	dropwise
9.00	–	dropwise	1	–
10.00	–	–	1	dropwise
11.00	–	10	3	10
12.00	–	–	dropwise	1
12.80	–	–	–	1

### The common procedure for preparation of CD imines **3**

Amino-β-CD **1** (≈0.2 mmol) and aldehyde **2** (up to 30 equiv) were refluxed in 100 mL of MeOH under argon overnight. The reaction was monitored by MS, and after the full conversion to imine, the solvent was evaporated. The unreacted aldehyde was extracted ten times with hexane (10 mL), and the product was dried in a Kugelrohr at 110 °C.

## Supporting Information

File 1 Synthesis and characterization data for compounds **3a**–**f**. Experimental data of time evolutions of integral of the imine group proton signal at 8.30 ppm in the acquired ^1^H NMR spectrum of the compound **3d** for various pH.
