# Supplementary material for: Cyclodextrin-based Schiff base pro-fragrances: Synthesis and release studies
Source: Beilstein J Org Chem. 2022 Sep 28;18:1346–54. doi: 10.3762/bjoc.18.140 (PMC9531562; doi:10.3762/bjoc.18.140)
Supplement: File 1 — Synthesis and characterization data for compounds 3a–f. Experimental data of time evolutions of integral of the imine group proton signal at 8.30 ppm in the acquired 1H NMR spectrum of the compound 3d for various pH. [file Beilstein_J_Org_Chem-18-1346-s001.pdf]

BEILSTEIN JOURNAL OF ORGANIC CHEMISTRY

Supporting Information

for

Cyclodextrin-based Schiff base pro-fragrances: Synthesis and release studies

Attila Palágyi, Jindřich Jindřich, Juraj Dian and Sophie Fourmentin

Beilstein J. Org. Chem. **2022,** *18,* 1346–1354. doi:10.3762/bjoc.18.140

Synthesis and characterization data for compounds 3a–f. Experimental data of time evolutions of integral of the imine group proton signal at 8.30 ppm in the acquired ^{1}H NMR spectrum of the compound 3d for various pH

License and Terms: This is a supporting information file under the terms of the Creative Commons Attribution License (https://creativecommons.org/licenses/by/4.0). Please note that the reuse, redistribution and reproduction in particular requires that the author(s) and source are credited and that individual graphics may be subject to special legal provisions.
The license is subject to the *Beilstein Journal of Organic Chemistry* terms and conditions: (https://www.beilstein-journals.org/bjoc/terms)

Contents

1. Synthesis and characterization data for compounds **3a–f** S2

2. Experimental data of the time evolutions of integral of the imine group proton signal at 8.30 ppm in the acquired ^{1}H NMR spectrum of the compound **3d** for pH = 1 and 2......S7

3. Experimental data of the time evolutions of integral of the imine group proton signal at 8.30 ppm in the acquired ^{1}H NMR spectrum of the compound **3d** for pH from 3 to 12.8. ....S7

1. Synthesis and characterization data for compounds **3a**-**3f**

6^{I}-Deoxy-6^{I}-(3-phenylallylidene)amino-β-cyclodextrin (3a)

Prepared from compound **1** (210.9 mg, 0.186 mmol) and *trans*-cinnamaldehyde (**2a**, 0.7 mL, 5.58 mmol) using the common procedure; 222.4 mg of yellowish powder **3a** afforded with the yield of 96%.

^{1}H NMR (600 MHz, DMSO-d_6): δ 8.44 (s, 1H, **H1'**), 8.07 (d, J = 8.6 Hz, 1H, **H7'**), 7.60 (d, J = 7.4 Hz, 2H, **H5'**), 7.52 (d, J = 7.4 Hz, 2H, **C6'**), 6.95 -6.91 (m, 1H, **H3'**), 6.41 (d, J = 18 Hz, 1H, **H2'**), 6.05 – 5.92 (m, 14H, **2,3-OH**), 4.90 – 4.81 (m, 7H, **H1**), 4.52 – 4.42 (m, 6H, **6-OH**), 3.88 – 3.30 (m, 42H, **H2**, **H3**, **H4**, **H5**, **H6**)

^{13}C NMR (125 MHz, DMSO-d_6): δ 162.50 (**C1'**), 137.34 (**C4'**), 129.31 (**C2'**), 129.13 (**C3'**), 128.71 (**C7'**), 127.69 (**C6'**), 127.58 (**C5'**), 102.56 – 102.25 (**C1^{I}**), 83.85 – 82.01 (**C4^{I}**), 73.64 – 70.95 (**C2^{I}**, **C3^{I}**, **C5^{I}**), 60.96 – 60.40 (**C6^{I}**)

For $C_{51}H_{77}NO_{34}$ calculated M_r: 1248.15, ESI-MS: *m/z* 1270 $[M + Na]^+$, $[\alpha]^{25}_{D}$ +103.4°

6^{I}-Deoxy-6^{I}-((3-(4-isopropylphenyl)-2-methylpropylidene)amino)-β-cyclodextrin (3b)

Prepared from compound **1** (211.0 mg, 0.186 mmol) and cyclamen aldehyde (**2b**, 1.18 mL, 5.58 mmol) using the common procedure; 200.7 mg of **3b** was afforded as a white powder with the yield of 83%.

^{1}H NMR (600 MHz, DMSO-d_6): δ 8.22 (s, 1H, **H1'**), 7.86 (d, J = 8.5 Hz, 2H, **H6'**), 7.67 (d, J = 8.4 Hz, 2H, **H5'**), 5.81 (bs, 14H, **2,3-OH**), 4.92 – 4.74 (m, 7H, **H1**), 4.62 – 4.24 (m, 6H, **6-OH**), 3.89 – 3.08 (m, 44H, **H2**, **H3**, **H4**, **H5**, **H6**, **H2'**, **H8'**), 2.73 – 2.70 (m, 2H, **H3'**), 1.38 (s, 6H, **H9'**), 0.93 (d, J = 6.6 Hz, 3H, **H10'**)

^{13}C NMR (125 MHz, DMSO-d_6): δ 162.28 (**C1'**), 132.79 (**C5'**), 130.53 (**C6'**), 103.14 – 102.51 (**C1^{I}**), 84.58 – 82.27 (**C4^{I}**), 74.27 – 71.56 (**C2^{I}**, **C3^{I}**, **C5^{I}**), 61.38 – 60.59 (**C6^{I}**), 56.68 (**C2'**), 56.21 (**C3'**), 34.94 (**C8'**), 27.39 (**C9'**), (**C10'**)

For $C_{55}H_{87}NO_{34}$ calculated M_r: 1306.27, ESI-MS: *m/z* 1328 $[M + Na]^+$, $[\alpha]^{25}_D$ +111.7°

6ᴵ-((3-(4-(*tert*-Butyl)phenyl)-2-methylpropylidene)amino)-6ᴵ-deoxy-β-cyclodextrin (3c)

Prepared from compound **1** (211.0 mg, 0.186 mmol) and lilial (**2c**, 0.84 mL, 5.58 mmol) using the common procedure; 223.6 mg of **3c** was afforded as a powder with the yield of 91%.

^{1}H NMR (600 MHz, DMSO-d_6): δ 8.47 (s, 1H, **H1’**), 7.22 (d, *J* = 8.1 Hz, 2H, **H6’**), 7.07 (d, *J* = 7.9 Hz, 2H, **H5’**), 5.81 (bs, 14H, **2,3-OH**), 4.85 – 4.64 (m, 7H, **H1**), 4.31 – 4.02 (m, 6H, **6-OH**), 3.77 – 3.03 (m, 42H, **H2**, **H3**, **H4**, **H5**, **H6**), 2.59 – 2.55 (m, 2H, **H3’**), 1.24 (s, 9H, **H9’**), 0.88 (d, *J* = 6.8 Hz, 3H, **H10’**), (1H, **H2’** peak is under DMSO peak at 2.54)

^{13}C NMR (125 MHz, DMSO-d_6): δ 167.10 (**C1’**), 162.06 (**C7’**), 139.74 (**C7’**), 137.27 (**C4’**), 129.19 (**C5’**), 125.26 (**C6’**), 129.99 (**C4’**), 103.05 – 102.45 (**C1ᴵ**), 85.25 – 81.90 (**C4ᴵ**), 75.86 – 81.90 (**C2ᴵ**, **C3ᴵ**, **C5ᴵ**), 61.13 – 60.04 (**C6ᴵ**), 41.86 (**C2’**), 39.27 (**C3’**), 34.94 (**C8’**), 32.25 (**C9’**), 31.88 (**C8’**)14.94 (**C10’**)

For $C_{56}H_{89}NO_{34}$ calculated M_r: 1320.30, ESI-MS: *m/z* 1342 $[M + Na]^+$, $[\alpha]^{25}_D$ +103.9°

6ᴵ-Benzylideneamino-6ᴵ-deoxy-β-cyclodextrin (3d)

Prepared from compound **1** (155.3 mg, 0.137 mmol) and benzaldehyde (**2d**, 0.28 mL, 2.74 mmol) using the common procedure; 144.3 mg of **3d** was afforded as a white powder with the yield of 86%.

^{1}H NMR (600 MHz, DMSO-d_6): δ 8.33 (s, 1H, **H1’**), 7.76 – 7.75 (m, 2H, **H3’**), 7.45 – 7.44 (m, 3H, **H4’**, **H5’**), 5.91 – 5.75 (m, 14H, **2,3-OH**), 4.96 – 4.80 (m, 7H, **H1**), 4.51 – 4.33 (m, 6H, **6-OH**), 3.82 – 3.32 (m, 42H, **H2**, **H3**, **H4**, **H5**, **H6**)

^{13}C NMR (125 MHz, DMSO-d_6): δ 162.49 (**C1'**), 130.92 (**C3'**), 128.93 (**C4'**), 128.42 (**C5'**), 102.60 – 101.98 (**C1^I**), 83.99 – 81.71 (**C4^I**), 73.64 – 72.42 (**C2^I**, **C3^I**, **C5^I**), 59.97 – 60.84 (**C6^I**)

For $C_{49}H_{75}NO_{34}$ calculated M_r: 1222.11, ESI-MS: *m/z* 1244 $[M + Na]^+$, $[\alpha]^{25}_D$ +103.4°

6^I-Deoxy-6^I-(4-methoxybenzylidene)amino-β-cyclodextrin (3e)

Prepared from compound **1** (211 mg, 0.186 mmol) and 4-anisaldehyde (**2e**, 0.35 mL, 2.80 mmol) using the common procedure; 191.1 mg of **3e** was afforded as a white powder with the yield of 82%.

^1H NMR (600 MHz, DMSO-d_6): δ 8.22 (s, 1H, **H1'**), 7.86 (d, J = 8.5 Hz, 2H, **H3'**), 7.11 (d, J = 8.4 Hz, 2H, **H4'**), 5.81 (bs, 14H, **2,3-OH**), 4.92 – 4.74 (m, 7H, **H1**), 4.64 – 4.24 (m, 6H, **6-OH**), 3.89 (s, 3H, **H6'**), 3.78 – 3.13 (m, 42H, **H2**, **H3**, **H4**, **H5**, **H6**)

^{13}C NMR (125 MHz, DMSO-d_6): δ 161.75 (**C1'**), 161.49 (**C5'**), 132.27 (**C3'**), 129.46 (**C2'**), 114.96 (**C4'**), 102.62 – 102.03 (**C1^I**), 84.06 – 81.74 (**C4^I**), 73.73 – 71.05 (**C2^I**, **C3^I**, **C5^I**), 60.85 – 59.98 (**C6^I**), 55.69 (**C6'**)

For $C_{50}H_{77}NO_{35}$ calculated M_r: 1252.14, ESI-MS: *m/z* 1274 $[M + Na]^+$, $[\alpha]^{25}_D$ +102.4°

6^I-Deoxy-6^I-(4-hydroxy-3-methoxybenzylidene)amino-β-cyclodextrin (3f)

Compound **1** (211 mg, 0.186 mmol) and vanillin (**2f**, 596 mg, 3.72 mmol) was refluxed in 100 mL of MeOH under argon overnight. The solvent was evaporated, and the remaining aldehyde was extracted ten times with toluene (10 mL) (instead of the hexane as in all other cases due to the lower hexane solubility of the aldehyde). The product was dried in Kugelrohr at 110 °C. The reaction gave 227.1 mg of yellow powder **3f** with the yield of 96%.

^1H NMR (600 MHz, DMSO-d_6): δ 8.17 (s, 1H, **H1'**), 7.37 (s, 1H, **H3'**), 7.17 (dd, J = 8.3, 2.1 Hz, 1H, **H6'**), 7.05 (d, J = 2.1 Hz, 1H, **H7'**), 5.19 (bs, 14H, **2,3-OH**), 4.94 –

4.84 (m, 7H, **H1**), 4.72 – 4.24 (m, 6H, **6-OH**), 3.96 – 3.32 (m, 42H, **H2**, **H3**, **H4**, **H5**, **H6**), 3.82 (s, 3H, **H8'**)

^{13}C NMR (125 MHz, DMSO-d_6): δ 162.27 (**C1'**), 151.37 (**C5'**), 148.47 (**C4'**), 130.30 (**C2'**), 123.45 (**C7'**), 117.33 (**C6'**), 110.37 (**C3'**), 102.66 – 102.13 (**C1^I**), 84.36 – 81.89 (**C4^I**), 73.70 – 71.09 (**C2^I**, **C3^I**, **C5^I**), 61.14 – 60.13 (**C6^I**), 55.20 (**C8'**)

For $C_{50}H_{77}NO_{36}$ calculated M_r: 1268.14, ESI-MS: m/z 1290 [M + Na]$^+$, $[\alpha]^{25}_D$ +88.6°

6^I-Deoxy-6^I-hexylideneamino-β-cyclodextrin (3g)

Prepared from compound **1** (211 mg, 0.186 mmol) and hexanal (**2g**, 0.46 mL, 3.72 mmol) using the common procedure; 201.3 mg of **3g** was afforded as a white crystalline with the yield of 89%.

^{1}H NMR (600 MHz, DMSO-d_6): δ 6.59 (s, 1H, **H1'**), 6.35 – 5.20 (m, 14H, **2,3-OH**), 5.05 – 4.86 (m, 7H, **H1**), 4.54 – 4.03 (m, 6H, **6-OH**), 3.82 – 2.95 (m, 42H, **H2**, **H3**, **H4**, **H5**, **H6**), 2.03 – 1.97 (m, 2H, **H2'**), 1.42 – 1.21 (m, 6H, **H3'**, **H4'**, **H5'**), 0.83 (s, 3H, **H6'**)

^{13}C NMR (125 MHz, DMSO-d_6): δ 156.21 (**C1'**), 102.83 – 102.18 (**C1^I**), 82.35 – 81.51 (**C4^I**), 73.53 – 72.26 (**C2^I**, **C3^I**, **C5^I**), 60.80 – 69.91 (**C6^I**), 36.87 (**C2'**), 31.70 (**C3'**), 25.71 (**C4'**), 22.43 (**C5'**), 14.38 (**C6'**)

For $C_{48}H_{81}NO_{34}$ calculated M_r: 1216.15, ESI-MS: *m/z* 1238 [M + Na]$^+$, $[\alpha]^{25}_D$ +105.1°

6^I-Deoxy-6^I-heptylideneamino-β-cyclodextrin (3h)

Prepared from compound **1** (211 mg, 0.186 mmol) and heptanal (**2h**, 0.53 mL, 3.72 mmol) using the common procedure; 221.1 mg of **3h** was afforded as a white powder with the yield of 97%.

^{1}H NMR (600 MHz, DMSO-d_6): δ 7.81 (d, *J* – 27.2 Hz, 1H, **H1'**), 6.33 – 5.72 (m, 14H, **2,3-OH**), 4.99 – 4.79 (m, 7H, **H1**), 4.49 (bs, 6H, **6-OH**), 3.59 – 3.27 (m, 42H, **H2**, **H3**,

H4, **H5**, **H6**), 2.24 – 2.17 (m, 2H, **H3’**), 2.00 – 1.89 (m, 2H, **H2’**), 1.44 – 1.08 (m, 6H, **H4’**, **H5’**, **H6’**), 0.88 (s, 3H, **H7’**)

^{13}C NMR (125 MHz, DMSO-d_6): δ 165.83 (**C1’**), 102.92 – 102.19 (**C1^{I}**), 82.40 – 81.67 (**C4^{I}**), 73.51 – 72.45 (**C2^{I}**, **C3^{I}**, **C5^{I}**), 60.72 – 59.99 (**C6^{I}**), 38.01 (**C2’**), 31.60 (**C3’**), 29.24 (**C4’**), 26.42 (**C5’**), 22.49 (**C6’**), 14.39 (**C7’**)

For $C_{49}H_{83}NO_{34}$ calculated M_r: 1230.18, ESI-MS: *m/z* 1252 $[M + Na]^+$, $[\alpha]^{25}_{D}$ +103.9°

6^{I}-Deoxy-6^{I}-(3,7-dimethylocta-2,6-dien-1-ylidene)amino-β-cyclodextrin (3i)

Prepared from compound 1 (211 mg, 0.186 mmol) and citral (**2i**, 1.00 mL, 5.58 mmol) using the common procedure; 188.9 mg of **3i** was afforded as a yellowish powder with the yield of 80%.

^{1}H NMR (600 MHz, DMSO-d_6): δ 8.48 (s, 1H, **H1’**), 6.16 – 5.83 (m, 14H, **2,3-OH**), 5.89 (bs, 1H, **H2’**), 5.17 – 5.09 (m, 1H, **H6’**) 4.86 – 4.69 (m, 7H, **H1**), 4.60 – 4.22 (m, 6H, **6-OH**), 3.83 – 3.09 (m, 42H, **H2**, **H3**, **H4**, **H5**, **H6**), 2.19 – 2.09 (m, 4H, **H4’**, **H5’**), 1.69 (s, 3H, **H9’**), 1.62 (s, 6H, **H8’**)

^{13}C NMR (125 MHz, DMSO-d_6): δ 162.80 (**C1’**), 126.26 – 123.64 (**C2’**, **C3’**, **C6’**, **C7’**), 102.59 – 102.09 (**C1^{I}**), 84.18 – 81.82 (**C4^{I}**), 73.91 – 71,04 (**C2^{I}**, **C3^{I}**, **C5^{I}**), 60.87 – 60.34 (**C6^{I}**), 26.78 – 26.16 (**C4’**, **C5’**), 25.97 (**C9’**), 18.05 (**C8’**)

For $C_{52}H_{85}NO_{34}$ calculated M_r: 1268.23, ESI-MS: *m/z* 1290 $[M + Na]^+$, $[\alpha]^{25}_{D}$ +94.6°

6^{I}-Deoxy-6^{I}-((5-methyltetrahydrofuran-2-yl)methylene)amino-β-cyclodextrin (3j)

Prepared from compound **1** (211 mg, 0.186 mmol) and 5-methylfurfural (**2j**, 0.56 mL, 5.58 mmol) using the common procedure; 191.2 mg of **3j** was afforded as a brownish powder with the yield of 83%.

^{1}H NMR (600 MHz, DMSO-d_6): δ 8.03 (s, 1H, **H1’**), 6.79 (d, J = 3.3 Hz, 1H, **H3’**), 6.22 (d, J = 3.1 Hz, 1H, **H4’**), 5.81 (bs, 14H, **2,3-OH**), 4.88 – 4.79 (m, 7H, **H1**), 4.43 (bs, 6H, **6-OH**), 3.88 – 3.28 (m, 42H, **H2**, **H3**, **H4**, **H5**, **H6**), 2.32 (s, 3H, **H6’**)

^{13}C NMR (125 MHz, DMSO-d_6): δ 162.58 (**C1’**), 151.45 (**C5’**), 115.99 (**C2’**), 111.24 (**C3’**), 108.66 (**C4’**), 102.64 – 102.19 (**C1**^{I}), 83.94 – 81.92 (**C4**^{I}), 73.73 – 72.47 (**C2**^{I}, **C3**^{I}, **C5**^{I}), 60.91 – 60.84 (**C6**^{I}), 13.93 (**C6’**)

For $C_{48}H_{75}NO_{35}$ calculated M_r: 1226.10, ESI-MS: m/z 1248[M + Na]$^{+}$, $[\alpha]^{25}_{D}$ +97.2°

2. Experimental data of the time evolutions of integral of the imine group proton signal at 8.30 ppm in the acquired ^{1}H NMR spectrum of the compound **3d** for pH = 1 and 2.

Table S1. Experimental data of the imine group proton signal for pH=1 and 2

pH=1		pH=2	
time	Integral intensity	time	Integral intensity
0:00:00	100	0:00:00	100
0:03:00	11	0:03:00	11
0:05:00	11	0:04:30	11
0:06:20	11	0:06:00	9
0:07:40	11	0:08:00	10
0:09:00	11	0:09:30	9
0:10:20	11	0:11:00	10
0:11:40	11	0:12:30	10
0:13:00	11	0:14:00	10
0:14:20	11	0:15:30	11
0:15:40	11	0:17:00	11
0:17:00	11	0:18:30	10
0:18:20	11	0:20:00	11
0:19:40	11	0:21:30	11
0:21:00	11	0:23:00	11

3. Experimental data of the time evolutions of integral of the imine group proton signal at 8.30 ppm in the acquired ^{1}H NMR spectrum of the compound **3d** for pH from 3 to 12.8.

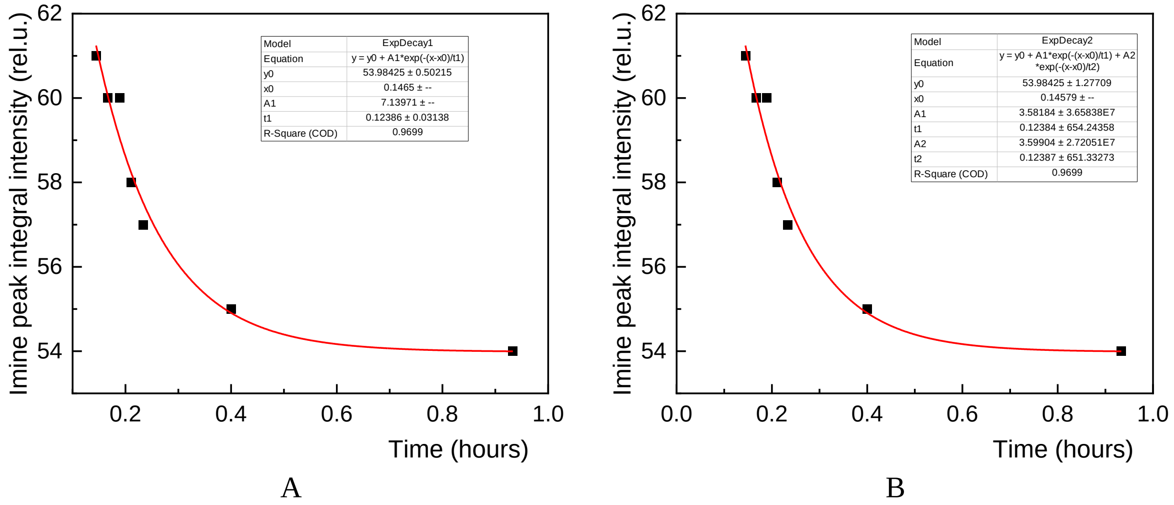

Figure S1: Fit of experimental data at pH = 3 by (A) – mono-exponential and (B) double-exponential function.

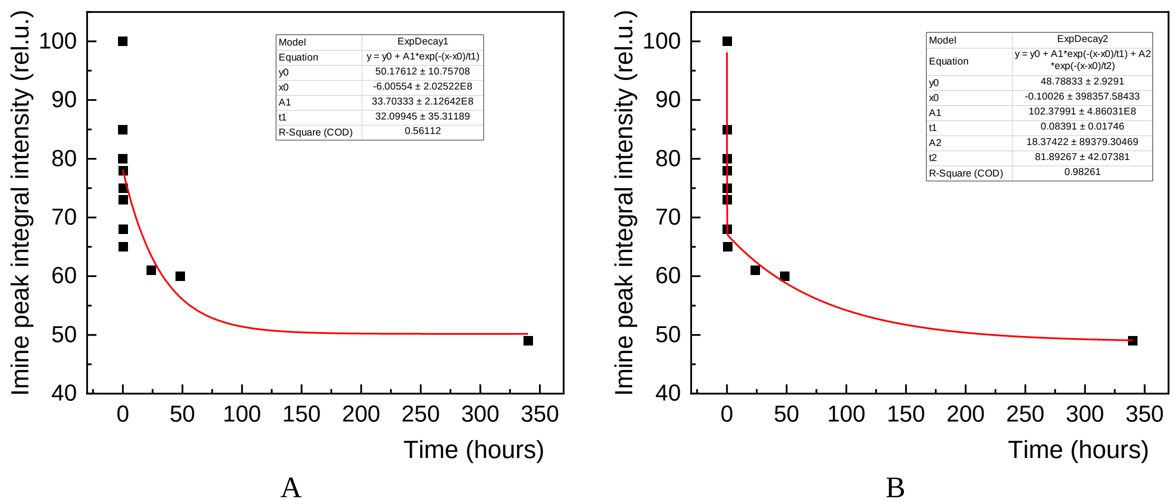

Figure S2: Fit of experimental data at pH = 4 by (A) – mono-exponential and (B) double-exponential function

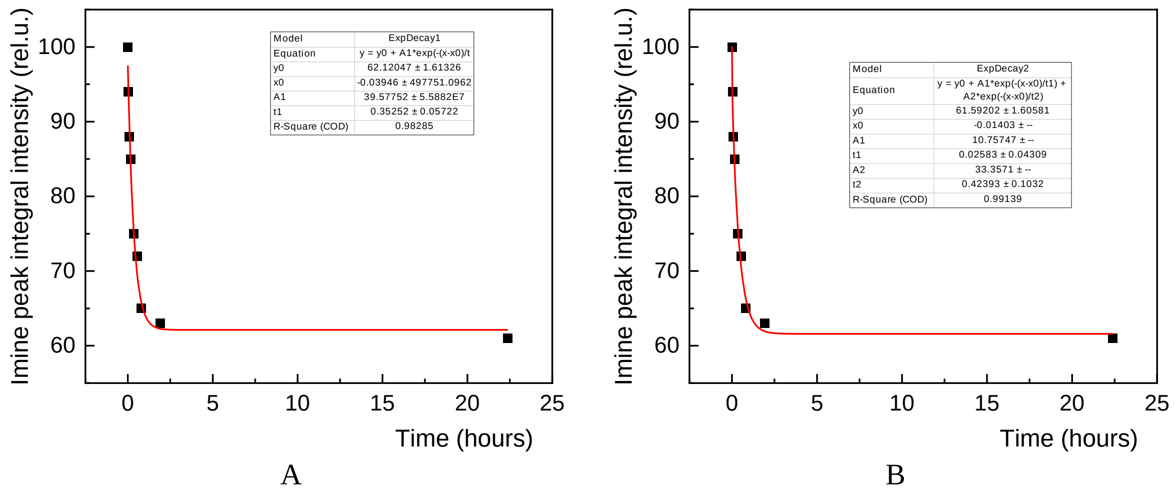

Figure S3: Fit of experimental data at pH = 5 by (A) – mono-exponential and (B) double-exponential function.

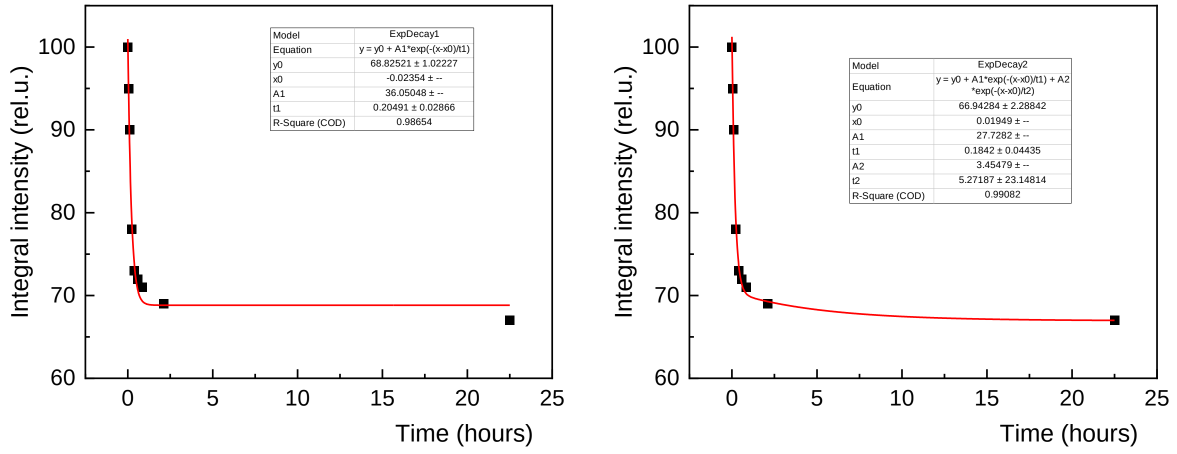

A B

Figure S4: Fit of experimental data at pH = 6 by (A) – mono-exponential and (B) double-exponential function.

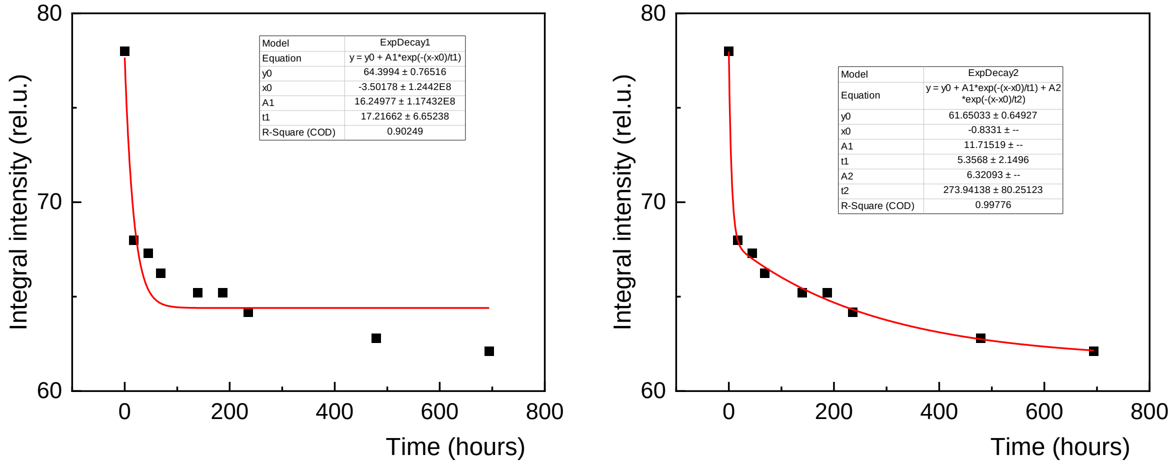

A B

Figure S5: Fit of experimental data at pH = 7 by (A) – mono-exponential and (B) double-exponential function.

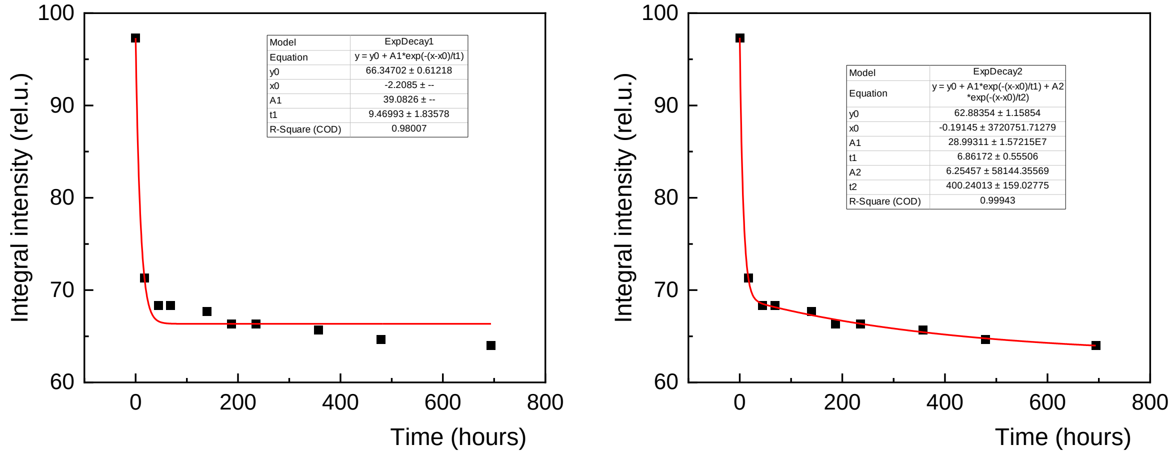

A B

Figure S6: Fit of experimental data at pH = 8 by (A) – mono-exponential and (B) double-exponential function.

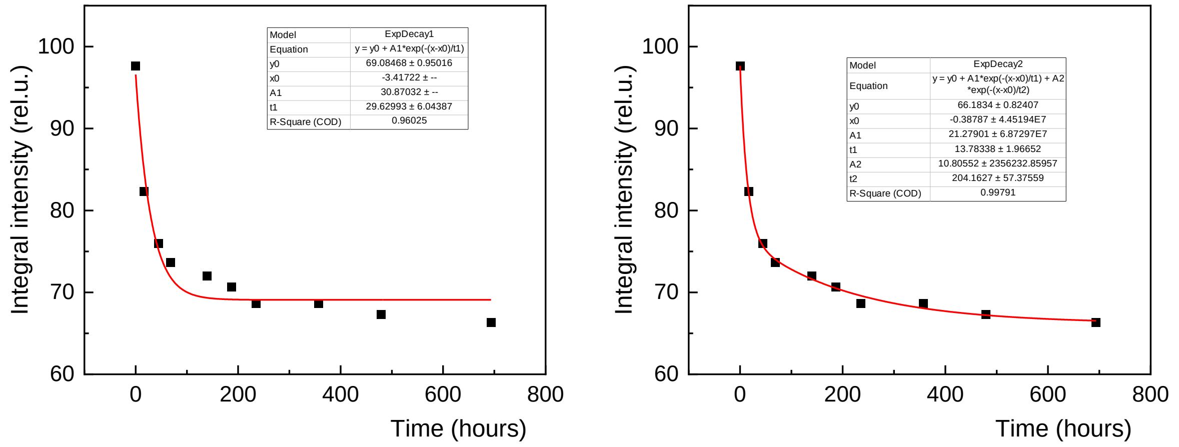

A B

Figure S7: Fit of experimental data at pH = 9 by (A) – mono-exponential and (B) double-exponential function.

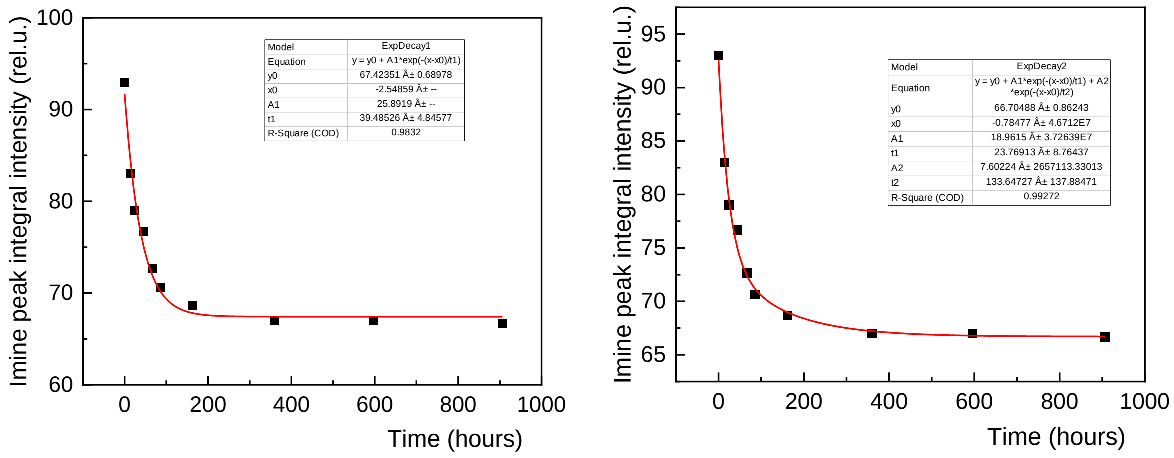

A B

Figure S8: Fit of experimental data at pH = 10 by (A) – mono-exponential and (B) double-exponential function.

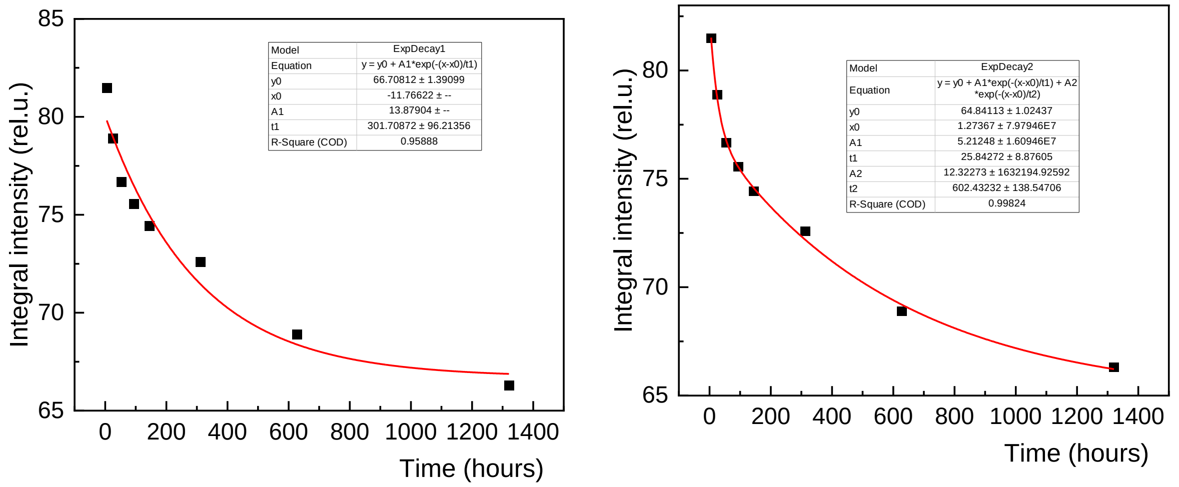

A B

Figure S9: Fit of experimental data at pH = 11 by (A) – mono-exponential and (B) double-exponential function.

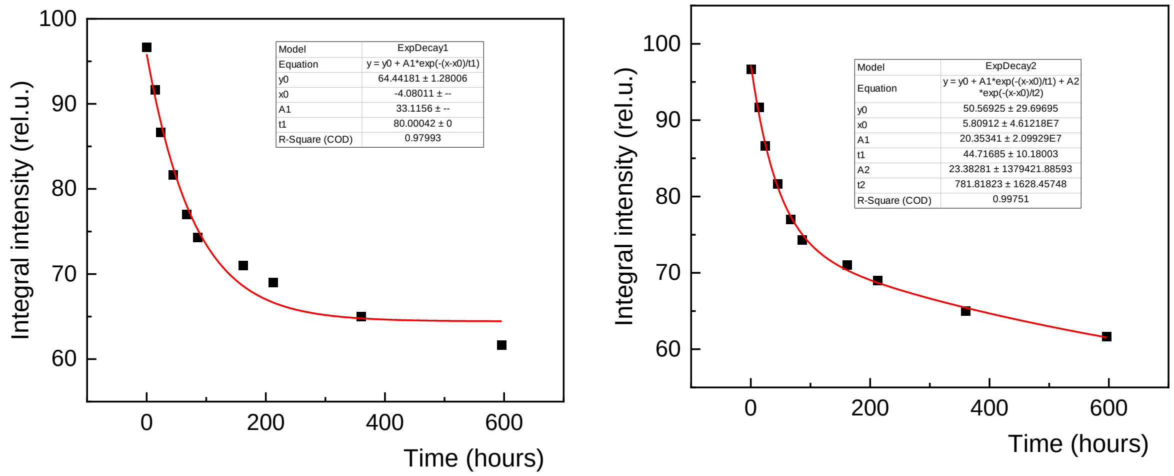

A B

Figure S10: Fit of experimental data at pH = 12 by (A) – mono-exponential and (B) double-exponential function.

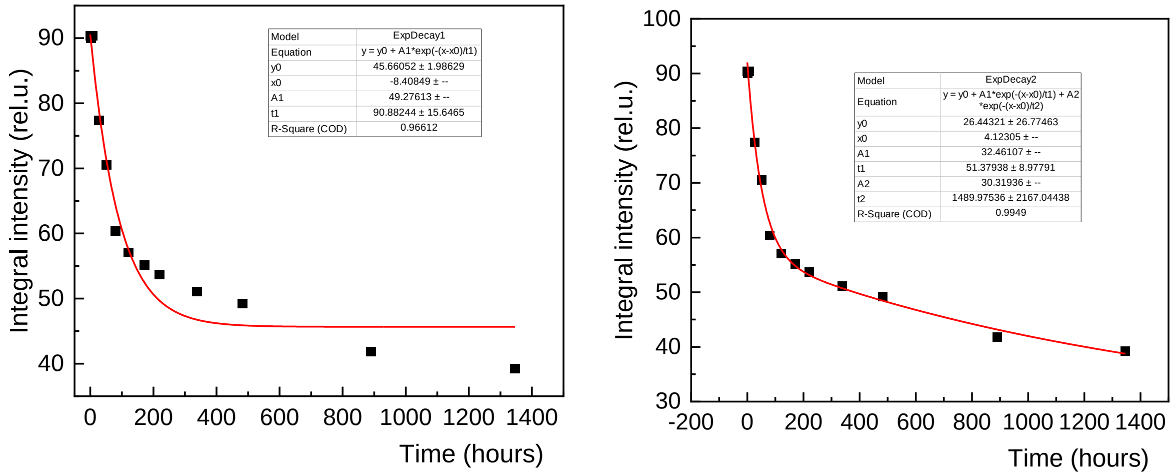

A B

Figure S11: Fit of experimental data at pH = 12.8 by (A) – mono-exponential and (B) double-exponential function.
